# A new species of *Ocotea* (Lauraceae) from Espírito Santo, Brazil

**DOI:** 10.3897/BDJ.14.e195965

**Published:** 2026-05-26

**Authors:** Lilian Silva Santos, Pedro Luís Rodrigues de Moraes

**Affiliations:** 1 Universidade Estadual Paulista (UNESP), Instituto de Biociências de Rio Claro, Programa de Pós-Graduação em Biologia Vegetal (Interunidades), Rio Claro, São Paulo, Brazil Universidade Estadual Paulista (UNESP), Instituto de Biociências de Rio Claro, Programa de Pós-Graduação em Biologia Vegetal (Interunidades) Rio Claro, São Paulo Brazil; 2 Universidade Estadual Paulista (UNESP), Instituto de Biociências de Rio Claro, Departamento de Biodiversidade, Bela Vista, 13506-900, Rio Claro, São Paulo, Brazil Universidade Estadual Paulista (UNESP), Instituto de Biociências de Rio Claro, Departamento de Biodiversidade, Bela Vista, 13506-900 Rio Claro, São Paulo Brazil

**Keywords:** Atlantic Rainforest, biodiversity, endemic species, Neotropics, taxonomy

## Abstract

**Background:**

*Ocotea* Aubl. is the largest genus amongst the Neotropical Lauraceae, estimated to have 469 recognised species in the Americas, 183 of them occurring in Brazil, 117 being endemic to the country. In the course of preparing a treatment of Lauraceae for the Flora of Espírito Santo, Brazil, an undescribed species of *Ocotea* was encountered. Its description and illustrations are presented here.

**New information:**

*Ocotea vervloetii* P.L.R.Moraes & L.S.Santos is a new species of *Ocotea* from the Atlantic Rainforest of south-eastern Brazil, collected in the Municipality of Santa Teresa, State of Espírito Santo. This new species probably belongs to the *Ocotea floribunda* group, since it bears relatively large male flowers ca. 7–8 mm diam., with receptacle flat, small, shallow, bowl-shaped and pistillode densely tomentose. Nevertheless, it is clearly different from other congeners in this group by presenting relatively short leaves, oblanceolate to obovate, elliptic, discolorous, coriaceous, with margin strongly revolute, adaxially glabrous to glabrescent, shiny, strongly bullate, abaxially sparsely to densely strigulose and papillose.

## Introduction

The new species presented here was first recognised as distinct in 2003, when the senior author came across two undetermined specimens of Lauraceae with male flowers in the Herbarium of Museu Mello Leitão (Santa Teresa, ES): one collected by Ludovic Kollmann in 2001; the other by Roxídio Vervloet in 2003. These specimens were matched with available keys to genera of Lauraceae (e.g. Mez (1889), Kostermans (1957), van der Werff (1991), Rohwer (1993), Alves et al. (2025)), in order to find out the correct genus; in all keys, the identification led to *Ocotea*. From April 2004 to June 2009, a study on the Lauraceae of the Santa Teresa microregion was conducted by Tiago Domingos Mouzinho Barbosa, then supervised by the senior author, which was later published by Barbosa et al. (2012). However, both the works by Quinet (2008), who revised *Ocotea* from south-eastern Brazil and Barbosa et al. (2012), did not analyse these specimens, which remained unidentified until 2022, when Alexandre Quinet erroneously annotated the duplicates at RB as belonging to *Persea*.

*Ocotea* Aubl. is a genus in the family Lauraceae belonging to the tribe Cinnamomeae Nees (Li et al. 2025). Currently, it encompasses about 511 species, 469 of them in the Neotropical Region and 42 species from Africa and neighbooring islands (34 in Madagascar) (Hurtado-Reveles and Ortiz-Rodriguez 2025, Li et al. 2025). POWO (2026) lists 518 accepted species, but several of them are taken as taxonomic synonymy in the list available in the Supplementary data of Li et al. (2025), which we are adopting here.

A synopsis of *Ocotea* was published by Rohwer (1986), who proposed its subdivision into 29 smaller informal groups, based on shared morphological features. Based on Rohwer’s key (Rohwer 1986) to the species groups of *Ocotea*, the new species will key to the *Ocotea floribunda* group, since it has unisexual flowers, stamens with locelli arranged in two superposed pairs, ovary ± reduced (in male flowers), but always with stigma, stamens of whorl III free, style and/or ovary hairy (in male flowers), leaf base attenuated to obtuse, leaves glabrous to moderately covered with appressed hairs abaxially, epidermis mostly visible and flower diameter over 4.5 mm.

Almost 25 years have passed since the first collection of the new species, which was only collected again two years later. Since then, it has apparently never been collected again, at least according to our searches for additional material available in the consulted herbaria. Although those specimens may be considered scanty for a description, their male flowers allow their identification in *Ocotea* and a probable placement in the *Ocotea floribunda* group. Moreover, the morphology of their leaves does not fit any of the known species of that group, nor any other accepted taxa of *Ocotea*, which leads us to determine their singularity and to describe them as a new species.

## Materials and methods

This study was based on literature review and morphological analysis of specimens deposited in the following Herbaria: ALCB, AWH, B, BAH, BHCB, BM, BR, CAP, CEN, CEPEC, CESJ, CGE, COAH, COI, COL, CVRD, EFC, ESA, ESAL, F, FLOR, FUEL, FURB, G, G-DC, GOET, GZU, HB, HBG, HBR, HCF, HRB, HRCB, HUEFS, HUESBVC, HUNEB, IAC, IAN, IBGE, ICN, INPA, IPA, K, KIEL, LE, LISU, M, MBM, MBML, MEL, MG, MO, NY, OXF, P, PEUFR, R, RB, RFA, SP, SPF, SPSF, UB, UEC, UESC, UFP, UPCB, VIC and VIES. Collections viewed only through digital images: A, C, CAS, CM, E, FI, GB, GH, HAL, HUFU, L, LD, LIL, LOJA, LSU, MA, MEXU, MICH, QCNE, S, TUB, U, UC, US, VEN, VT, W and WIS (acronyms according to Thiers (2026)) through the Global Biodiversity Information Facility - GBIF (https://www.gbif.org/), the JSTOR Global Plants database (https://plants.jstor.org/), specimen records at the Harvard University Herbaria (https://kiki.huh.harvard.edu/databases/specimen_index.html), specimen data from the Consortium of California Herbaria (https://www.cch2.org/portal/collections/search/index.php), dataportal of The University and Jepson Herbaria – University of California, Berkeley (https://webapps.cspace.berkeley.edu/ucjeps/publicsearch/publicsearch/), the Mid-Atlantic Herbaria Consortium portal (https://midatlanticherbaria.org/portal/collections/index.php), the Royal Botanic Garden Edinburgh herbarium catalogue (https://www.rbge.org.uk/), digital specimen images from the FI Herbaria (https://parlatore.msn.unifi.it/types/search.php), database of Herbario Nacional de México (MEXU) (https://ibdata4.ib.unam.mx/web/), the SEINet portal network (https://swbiodiversity.org/seinet/index.php), *species*Link Network (https://specieslink.net/), Naturalis Bioportal (https://bioportal.naturalis.nl/en), Sweden’s Virtual Herbarium (https://herbarium.emg.umu.se/), the plant collections of the Smithsonian Institution (https://collections.nmnh.si.edu/search/botany/), the Reflora - Virtual Herbarium (https://reflora.jbrj.gov.br/reflora/herbarioVirtual/) and the JACQ database (https://jacq.org/database). The botanical drawings of Lauraceae by Prof. Jens G. Rohwer, available at FUNDus! (Collection Portal of the University of Hamburg – https://www.fundus.uni-hamburg.de/en/search/find?q=lauraceae), were also consulted. Photographs of floral structures of the new species were obtained with a stereomicroscope (Leica M80), equipped with a camera (Leica IC80 HD), using the software LAS version 4.3.0.

GeoCAT (Bachman et al. 2011) was used to estimate the area of occupancy (AOO), with the AOO based on 4 km^2^ cells. The conservation assessment followed IUCN (2024) criteria. The occurrence map was built using QGIS software, version 3.34 (QGIS 2025).

## Taxon treatments

### Ocotea vervloetii

P.L.R.Moraes & L.S.Santos sp. nov.

4812627B-C966-546B-A3A1-26860454BE2E

77380715-1

#### Materials

- **Type status:** Holotype. **Occurrence:** catalogNumber: MBML020694; recordNumber: 2362; recordedBy: R.R. Vervloet; sex: male; reproductiveCondition: bud, fl.; occurrenceID: 272F8A07-9D33-5420-9AAB-DA0BC9B9792B; **Taxon:** kingdom: Plantae; phylum: Tracheophyta; class: Magnoliopsida; order: Laurales; family: Lauraceae; genus: Ocotea; specificEpithet: vervloetii; scientificNameAuthorship: P.L.R.Moraes & L.S.Santos; **Location:** continent: South America; country: Brazil; stateProvince: Espírito Santo; municipality: Santa Teresa; locality: Reserva Biológica Augusto Ruschi; verbatimElevation: 800 m; verbatimLatitude: 19°54'27"S; verbatimLongitude: 40°33'11"W; decimalLatitude: -19.907500; decimalLongitude: -40.553056; **Event:** year: 2003; month: 5; day: 7
- **Type status:** Isotype. **Occurrence:** catalogNumber: ESAL 23800; recordNumber: 2362; recordedBy: R.R. Vervloet; sex: male; reproductiveCondition: bud, fl.; occurrenceID: 079ED574-205C-5BAF-84E9-65C0170819C0; **Taxon:** kingdom: Plantae; phylum: Tracheophyta; class: Magnoliopsida; order: Laurales; family: Lauraceae; genus: Ocotea; specificEpithet: vervloetii; scientificNameAuthorship: P.L.R.Moraes & L.S.Santos; **Location:** continent: South America; country: Brazil; stateProvince: Espírito Santo; municipality: Santa Teresa; locality: Reserva Biológica Augusto Ruschi; verbatimElevation: 800 m; verbatimLatitude: 19°54'27"S; verbatimLongitude: 40°33'11"W; decimalLatitude: -19.907500; decimalLongitude: -40.553056; **Event:** year: 2003; month: 5; day: 7
- **Type status:** Isotype. **Occurrence:** catalogNumber: HRCB084857; recordNumber: 2362; recordedBy: R.R. Vervloet; sex: male; reproductiveCondition: bud, fl.; occurrenceID: A8965CFE-00DB-5EDF-B02D-C3B2FA2DF78B; **Taxon:** kingdom: Plantae; phylum: Tracheophyta; class: Magnoliopsida; order: Laurales; family: Lauraceae; genus: Ocotea; specificEpithet: vervloetii; scientificNameAuthorship: P.L.R.Moraes & L.S.Santos; **Location:** continent: South America; country: Brazil; stateProvince: Espírito Santo; municipality: Santa Teresa; locality: Reserva Biológica Augusto Ruschi; verbatimElevation: 800 m; verbatimLatitude: 19°54'27"S; verbatimLongitude: 40°33'11"W; decimalLatitude: -19.907500; decimalLongitude: -40.553056; **Event:** year: 2003; month: 5; day: 7
- **Type status:** Isotype. **Occurrence:** catalogNumber: RB01465574; recordNumber: 2362; recordedBy: R.R. Vervloet; sex: male; reproductiveCondition: bud, fl.; occurrenceID: 0859FEC9-B143-5D3D-8457-887C52A8BE28; **Taxon:** kingdom: Plantae; phylum: Tracheophyta; class: Magnoliopsida; order: Laurales; family: Lauraceae; genus: Ocotea; specificEpithet: vervloetii; scientificNameAuthorship: P.L.R.Moraes & L.S.Santos; **Location:** continent: South America; country: Brazil; stateProvince: Espírito Santo; municipality: Santa Teresa; locality: Reserva Biológica Augusto Ruschi; verbatimElevation: 800 m; verbatimLatitude: 19°54'27"S; verbatimLongitude: 40°33'11"W; decimalLatitude: -19.907500; decimalLongitude: -40.553056; **Event:** year: 2003; month: 5; day: 7
- **Type status:** Isotype. **Occurrence:** catalogNumber: UEC115305; recordNumber: 2362; recordedBy: R.R. Vervloet; sex: male; reproductiveCondition: bud, fl.; occurrenceID: 59B25EC3-9FC8-5CC8-9408-763DD75DF2E5; **Taxon:** kingdom: Plantae; phylum: Tracheophyta; class: Magnoliopsida; order: Laurales; family: Lauraceae; genus: Ocotea; specificEpithet: vervloetii; scientificNameAuthorship: P.L.R.Moraes & L.S.Santos; **Location:** continent: South America; country: Brazil; stateProvince: Espírito Santo; municipality: Santa Teresa; locality: Reserva Biológica Augusto Ruschi; verbatimElevation: 800 m; verbatimLatitude: 19°54'27"S; verbatimLongitude: 40°33'11"W; decimalLatitude: -19.907500; decimalLongitude: -40.553056; **Event:** year: 2003; month: 5; day: 7
- **Type status:** Paratype. **Occurrence:** catalogNumber: BHCB 96128; recordNumber: 3822; recordedBy: L.J.C. Kollmann; sex: male; reproductiveCondition: bud, fl.; occurrenceID: E1EB1441-6627-56A5-8CAC-12B5C9A11DEC; **Taxon:** kingdom: Plantae; phylum: Tracheophyta; class: Magnoliopsida; order: Laurales; family: Lauraceae; genus: Ocotea; specificEpithet: vervloetii; scientificNameAuthorship: P.L.R.Moraes & L.S.Santos; **Location:** continent: South America; country: Brazil; stateProvince: Espírito Santo; municipality: Santa Teresa; locality: Valsugana Velha; verbatimElevation: 750 m; verbatimLatitude: 19°57'01"S; verbatimLongitude:  40°34'54"W; decimalLatitude: -19.950278 ; decimalLongitude: -40.581667; **Event:** year: 2001; month: 6; day: 6
- **Type status:** Paratype. **Occurrence:** catalogNumber: ESAL 23782; recordNumber: 3822; recordedBy: L.J.C. Kollmann; sex: male; reproductiveCondition: bud, fl.; occurrenceID: CA12BEBA-9B51-5B39-8D20-C5E4F2E7B331; **Taxon:** kingdom: Plantae; phylum: Tracheophyta; class: Magnoliopsida; order: Laurales; family: Lauraceae; genus: Ocotea; specificEpithet: vervloetii; scientificNameAuthorship: P.L.R.Moraes & L.S.Santos; **Location:** continent: South America; country: Brazil; stateProvince: Espírito Santo; municipality: Santa Teresa; locality: Valsugana Velha; verbatimElevation: 750 m; verbatimLatitude: 19°57'01"S; verbatimLongitude:  40°34'54"W; decimalLatitude: -19.950278 ; decimalLongitude: -40.581667; **Event:** year: 2001; month: 6; day: 6
- **Type status:** Paratype. **Occurrence:** catalogNumber: HRCB084858; recordNumber: 3822; recordedBy: L.J.C. Kollmann; sex: male; reproductiveCondition: bud, fl.; occurrenceID: 868655EE-D40F-5CE1-AFAD-C318DD63060A; **Taxon:** kingdom: Plantae; phylum: Tracheophyta; class: Magnoliopsida; order: Laurales; family: Lauraceae; genus: Ocotea; specificEpithet: vervloetii; scientificNameAuthorship: P.L.R.Moraes & L.S.Santos; **Location:** continent: South America; country: Brazil; stateProvince: Espírito Santo; municipality: Santa Teresa; locality: Valsugana Velha; verbatimElevation: 750 m; verbatimLatitude: 19°57'01"S; verbatimLongitude:  40°34'54"W; decimalLatitude: -19.950278 ; decimalLongitude: -40.581667; **Event:** year: 2001; month: 6; day: 6
- **Type status:** Paratype. **Occurrence:** catalogNumber: MBML014500; recordNumber: 3822; recordedBy: L.J.C. Kollmann; sex: male; reproductiveCondition: bud, fl.; occurrenceID: 4FBE01B7-0BC3-514A-96C0-7D775BD5ADF7; **Taxon:** kingdom: Plantae; phylum: Tracheophyta; class: Magnoliopsida; order: Laurales; family: Lauraceae; genus: Ocotea; specificEpithet: vervloetii; scientificNameAuthorship: P.L.R.Moraes & L.S.Santos; **Location:** continent: South America; country: Brazil; stateProvince: Espírito Santo; municipality: Santa Teresa; locality: Valsugana Velha; verbatimElevation: 750 m; verbatimLatitude: 19°57'01"S; verbatimLongitude:  40°34'54"W; decimalLatitude: -19.950278 ; decimalLongitude: -40.581667; **Event:** year: 2001; month: 6; day: 6
- **Type status:** Paratype. **Occurrence:** catalogNumber: RB01465573; recordNumber: 3822; recordedBy: L.J.C. Kollmann; sex: male; reproductiveCondition: bud, fl.; occurrenceID: E55B90F9-1C0A-5B15-A7B2-FC68657F543D; **Taxon:** kingdom: Plantae; phylum: Tracheophyta; class: Magnoliopsida; order: Laurales; family: Lauraceae; genus: Ocotea; specificEpithet: vervloetii; scientificNameAuthorship: P.L.R.Moraes & L.S.Santos; **Location:** continent: South America; country: Brazil; stateProvince: Espírito Santo; municipality: Santa Teresa; locality: Valsugana Velha; verbatimElevation: 750 m; verbatimLatitude: 19°57'01"S; verbatimLongitude:  40°34'54"W; decimalLatitude: -19.950278 ; decimalLongitude: -40.581667; **Event:** year: 2001; month: 6; day: 6

#### Description

**Figure 1.**
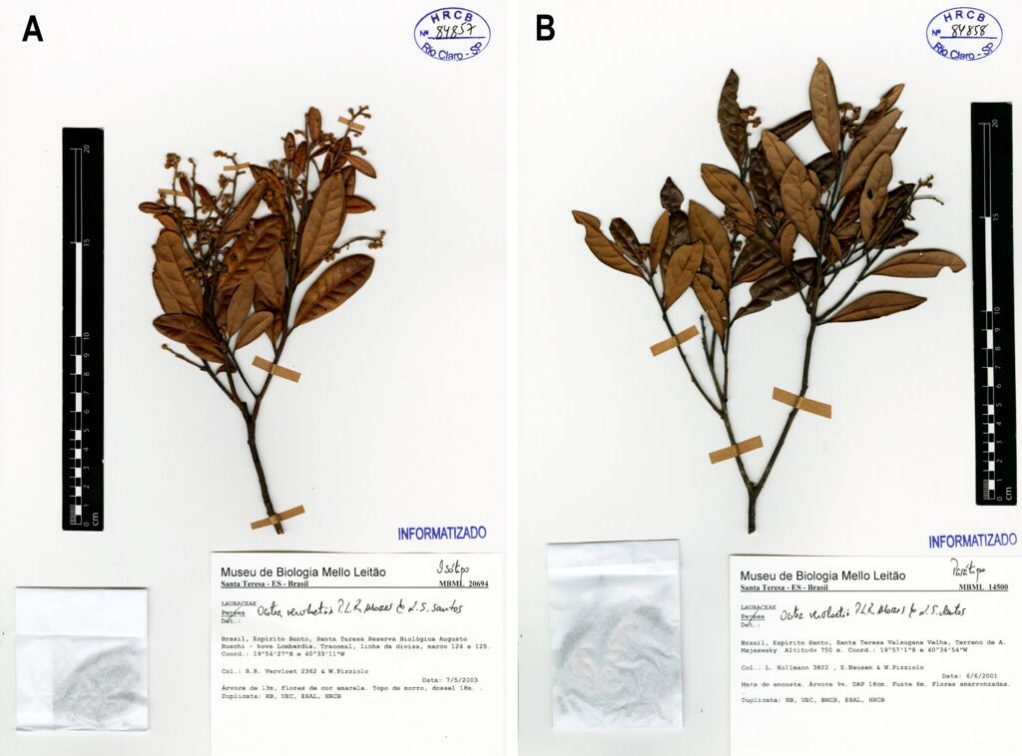
*Ocotea vervloetii* P.L.R.Moraes & L.S.Santos. **A** Isotype, *R.R. Vervloet & W. Pizziolo 2362* (HRCB084857); **B** Paratype, *L.J.C. Kollmann et al. 3822* (HRCB084858).

**Figure 2.**
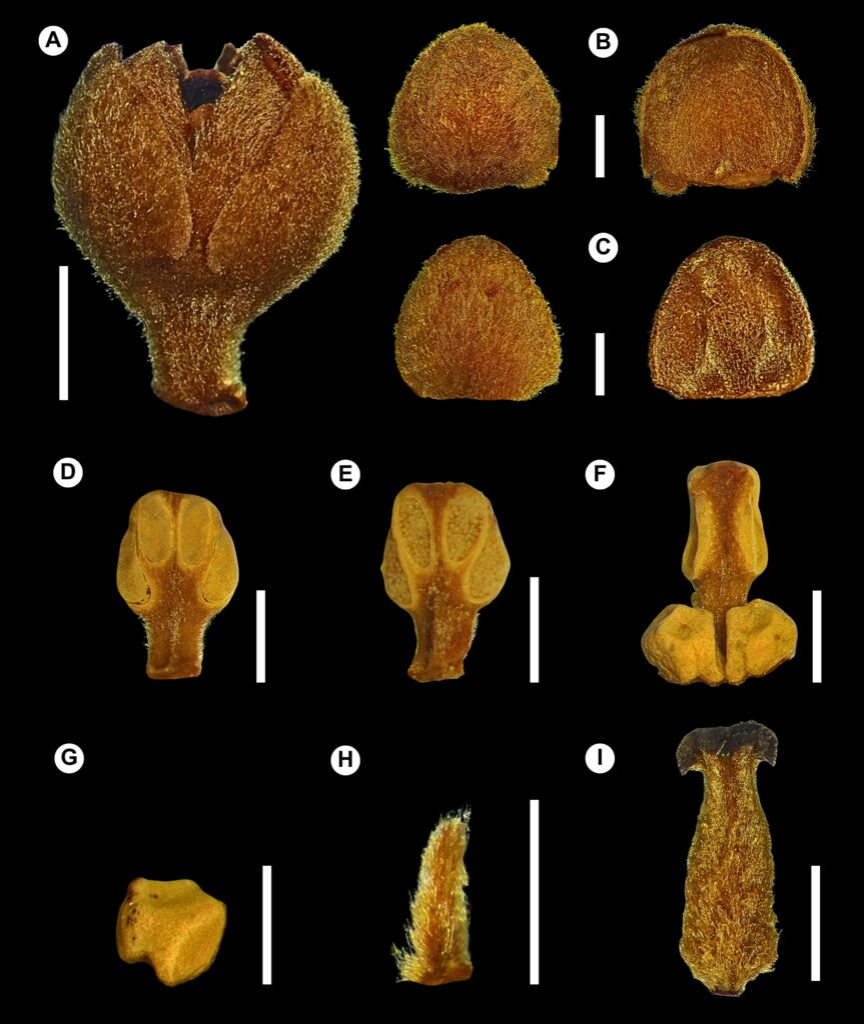
*Ocotea vervloetii* P.L.R.Moraes & L.S.Santos. **A** Flower; **B** Outer tepals, abaxial and adaxial surfaces; **C** Inner tepals, abaxial and adaxial surfaces; **D** Stamen of whorl I; **E** Stamen of whorl II; **F** Stamen of whorl III with glands; **G** Gland; **H** Staminode of whorl IV; **I** Pistillode. From: *R.R. Vervloet & W. Pizziolo 2362*. Bars: 2 mm (A), 1 mm (B–I).

**Figure 3.**
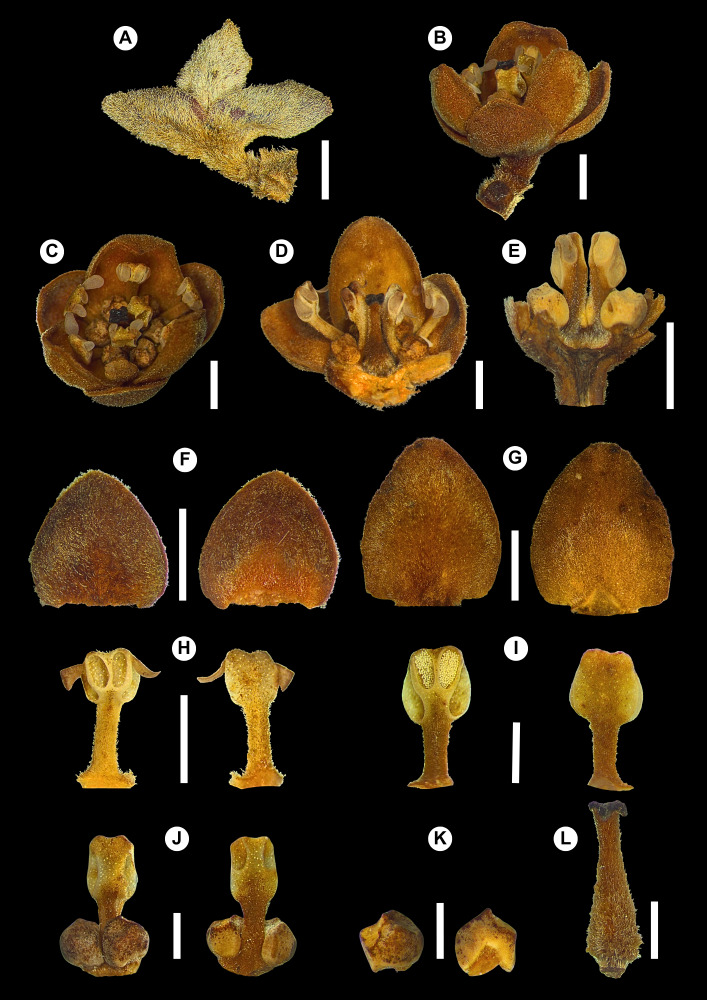
*Ocotea vervloetii* P.L.R.Moraes & L.S.Santos. **A** Dried flower; **B** Rehydrated flower, frontal view; **C** Rehydrated flower, top view; **D** Dissected flower with tepals and other floral parts partially removed, showing stamens of whorls I and III and pistillode; **E** Dissected flower showing stamens of whorl III with glands and the shallow receptacle; **F** Outer tepals, abaxial and adaxial surfaces; **G** Inner tepals, abaxial and adaxial surfaces; **H** Stamen of whorl I, adaxial and abaxial surfaces; **I** Stamen of whorl II, adaxial and abaxial surfaces; **J** Stamen of whorl III, abaxial and adaxial surfaces; **K** Gland, abaxial and adaxial surfaces; **L** Pistillode. From: *L.J.C. Kollmann et al. 3822*. Bars: 2 mm (A–H), 1 mm (I–L).

**Trees**, 9–13 m tall. **Branchlets** cylindrical, fissured, non-lenticellate, glabrous to sparsely strigulose (hairs short, appressed and straight); young branches slightly angular, sparsely to densely strigulose (hairs short, appressed, straight to wavy, rarely erect to ascending or long). **Terminal buds** densely strigulose to sericeous, trichomes yellowish (hairs short, appressed, straight). **Petiole** 0.3–0.7 cm long, 2.8–4.5 mm thick, slightly canaliculate above, glabrescent to sparsely strigulose (hairs short, appressed to ascending, straight to wavy). **Leaves** alternate, discolorous, coriaceous, 2.3–7.1 cm × 0.8–2.4 cm, oblong-obovate to oblanceolate, base acute to cuneate, symmetric, apex acute to short acuminate, rarely rounded, margin strongly revolute; adaxial surface dark brown [in sicco], shiny, strongly bullate, glabrous to glabrescent (hairs short, appressed, straight to wavy) along the mid-rib; mid-rib and secondary veins impressed; abaxial surface light brown, sparsely to densely strigulose (hairs short, appressed, straight to wavy), papillose; mid-rib and secondary veins prominent, 6–8 secondary veins on each side, diverging at an angle of 41°–80° from the mid-rib; reticulation dense, conspicuous; venation pinnate, eucamptodromous-brochidodromous; domatia absent. **Inflorescences** subterminal to axillary, paniculate-cymose, 0.6–8.5 cm long, shorter to longer than leaves, usually few- to moderate-flowered, with up to 35 flowers, densely strigulose to tomentose (hairs short to long, appressed to ascending, rarely erect, wavy to curly), yellowish. **Male flowers** densely strigulose to tomentose (hairs short to long, appressed to ascending, rarely erect, wavy to curly), yellowish, ca. 7–8 mm diam., pedicel 0.8–1.8 mm long; tepals 6, subequal (inner slightly larger), ovate to triangular, apex obtuse to rarely rounded; outer tepals, 2.5–4 mm × 2.4–4.1 mm, densely tomentose to sericeous (hairs short, appressed to erect, straight to curly) on both surfaces; inner tepals 2.4–5 mm × 2.5–4.1 mm, densely tomentose to sericeous (hairs short, appressed to erect, straight to curly) on both surfaces, spreading at anthesis; fertile stamens 9, 4-locular; the outer six (whorls I and II) 1.8–2.8 mm long, introrse, anthers ovoid to trapeziform, rarely elliptic, 1.2–1.3(1.5) mm long, strigose to pubescent, filaments (0.7)1–1.6 mm long, sparsely to densely strigose to pubescent (hairs short, ascendant, straight to wavy); the inner three stamens (whorl III) 2–2.9 mm long, anthers rectangular to trapeziform, 1.2–1.5 mm long, glabrescent to strigose, upper sporangia introrse to introrse-latrorse, lower pair extrorse-latrorse, filaments 0.9–1.4 mm long, sparsely to densely strigose (hairs short, appressed to ascendant, straight to wavy); glands irregularly polyhedric, 0.7–1.1 mm diam., sessile, adnate at base of filament; staminodes (whorl IV) 3 or absent, filiform to stipitiform, or stipitiform with the tip slightly cordiform to triangular, 0.6–1 mm long, densely tomentose to lanate (hairs short to long, ascending, straight to wavy); pistillode stipitiform to ellipsoid, slender, not swollen at the base, (2.3)2.5–3.1 mm long, densely tomentose (hairs short to long, ascending to erect, straight to wavy), sterile ovary ovoid, 1–1.5 mm × 0.8–1 mm, style 1.4–1.7 mm, stigma conspicuous; receptacle flat, small, shallow, bowl-shaped, 0.2–0.5 mm × 1.7–2.6 mm, densely pubescent inside. **Female flowers** unknown. **Fruits** unknown (Figs 1, 2, 3).

#### Diagnosis

Amongst the Brazilian Lauraceae from the Atlantic Rainforest domain, *Ocotea vervloetii* is very distinctive by its relatively short petioles, 0.3–0.7 cm long, short leaves, 2.3–7.1 cm × 0.8–2.4 cm, oblong-obovate to oblanceolate, discolorous, coriaceous, margin strongly revolute, adaxially glabrous to glabrescent, shiny, strongly bullate, abaxially sparsely to densely strigulose, papillose; inflorescences densely strigulose to tomentose; male flowers ca. 7–8 mm diam., spreading at anthesis, receptacle flat, small, shallow, bowl-shaped, 0.2–0.5 mm × 1.7–2.6 mm, densely pubescent inside, tepals subequal, staminodes filiform to stipitiform or lacking, pistillode 2.3–3.1 mm long, slender, subelliptic, densely tomentose.

#### Etymology

This species is named in homage to Roxísio Vervloet Romagna, a biologist born in Santa Teresa and a prolific collector of many plants in that region.

#### Distribution

**Figure 4.**
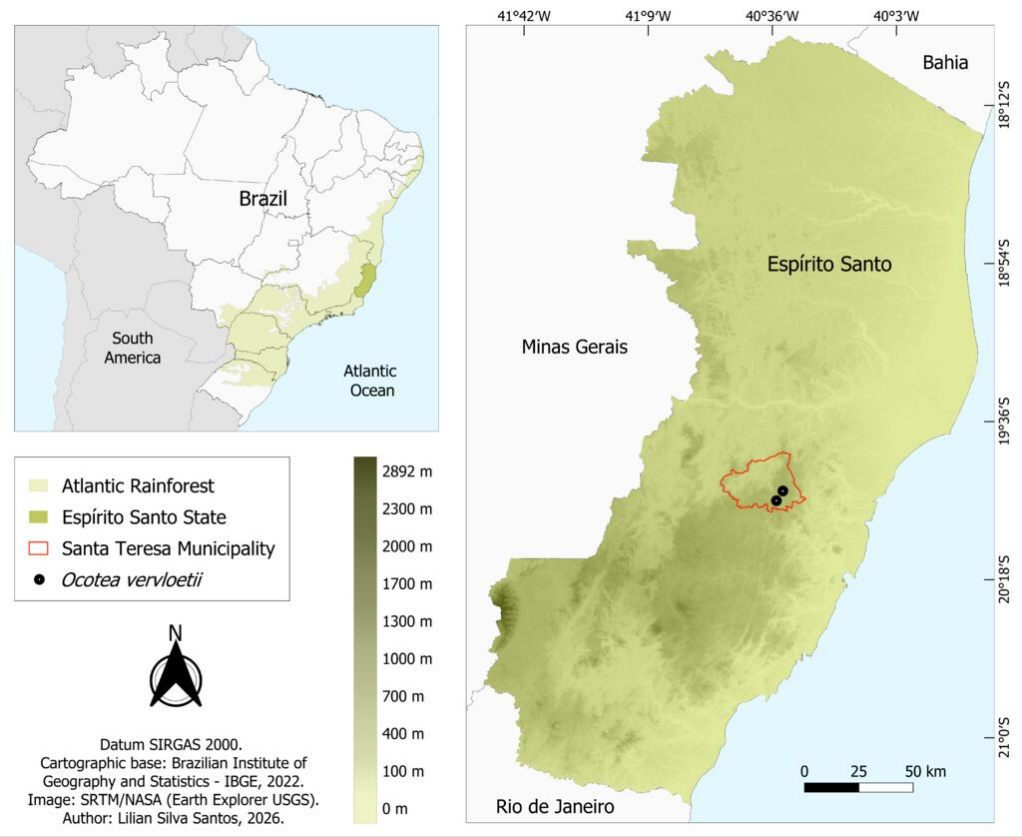
Geographical distribution of *Ocotea vervloetii* P.L.R.Moraes & L.S.Santos.

*Ocotea vervloetii* was collected from only two sites in the Municipality of Santa Teresa: one near Linha da Divisa, in Nova Lombardia, within the Augusto Ruschi Biological Reserve (19°54'27"S, 40°33'11"W, ca. 800 m); and another on a private property in the Valsugana Velha region (19°57'01"S, 40°34'54"W, ca. 750 m) (Fig. 4). Both sites, as well as the entire Espírito Santo State, are within the Atlantic Rainforest domain, in the Montane Dense Ombrophilous Forest phytophysiognomy (IBGE 2012). The area of occurrence belongs to the Paraíba do Sul Complex, dating to the Proterozoic. Its lithology is composed of metamorphic rocks of the Araçuaí-Rio Doce Orogeny, within the Mantiqueira Province, a mountainous region formed by geotectonic processes (Heilbron et al. 2004, BDiA 2025). The Nova Lombardia Region lies within the geomorphological unit of the “Planalto da Pedra Azul Capixaba”, mainly composed of hills and low mountains, with elevations ranging from 700 to 1,000 m. The Valsugana Velha Region is also characterised by hilly and mountainous relief. The “Morros e Montanhas do Centro-Sul Capixaba” unit is situated between the “Planalto da Pedra Azul Capixaba” and the “Colinas e Maciços Costeiros Capixabas” units, with elevations ranging from 20 to 1,300 m (BDiA 2025). The soil in both areas is classified as “CXbd - Cambissolo Háplico Tb Distrófico” (Haplic Cambisol (Eutric/Distric) Tb), according to the Brazilian Soil Classification System (SiBCS). It has a clayey texture, may be stony or non-stony and occurs in mountainous and escarped terrains (IBGE 2015, Cunha et al. 2016, BDiA 2025, Santos et al. 2025).

#### Conservation

To date, *Ocotea vervloetii* is known from only two individuals recorded at two sites 5.6 km apart in a straight line, one of them located within the Augusto Ruschi Biological Reserve. This restricted area and small number of samples resulted in an Area of Occupancy (AOO) of 8 km², leading to an assessment as Critically Endangered [CR], according to the IUCN Red List Categories and Criteria (IUCN 2024). However, we consider that the species’ distribution must be much wider throughout the mountainous microregion of Santa Teresa and that the small sampling of the species might be a reflection of the still existing undersampling of tree species in the region. Despite the ca. 1,083 distinct records of Lauraceae in the Municipality of Santa Teresa (*species*Link Network – https://specieslink.net/: speciesLink network, Abr 04, 2026 14:18, specieslink.net/search / (norm_stateprovince:((espírito santo))) AND (county:((Santa Teresa))) AND ((family:Lauraceae))), some areas still remain poorly sampled, most likely due to the difficulty of accessing densely forested and mountainous regions. The main threats to the species are related to habitat destruction. In 2014, only 32.1% of Santa Teresa’s native forest cover remained preserved. Pasture accounted for 14.8% of the Municipality’s land use, along with anthropogenised areas dedicated to agricultural activities, such as coffee and *Eucalyptus* plantations (Espírito Santo 2018). Nevertheless, currently there is no recognised threat to either the sites or trees of the new species, nor evidence of decline in AOO or the habitat. In short, considering that the microregion Santa Teresa remains under-collected concerning the trees, our understanding of their distribution is still insufficient. Therefore, *Ocotea vervloetii*’s risk of extinction is provisionally assessed as Data Deficient [DD] (IUCN 2024), since we do not have appropriate data on its abundance or distribution.

#### Phenology

The flowering specimens were collected in May and June.

#### Taxonomic notes

As mentioned above, *Ocotea vervloetii* most likely is related to the *O. floribunda* group *sensu* Rohwer (1986), based on his key to the species groups in *Ocotea* and for its combination of features like the large male flowers ca. 7–8 mm diam., receptacle small, shallow, bowl-shaped and pistillode densely tomentose, which also point to a placement in that group as circumscribed by Rohwer (1986). Species of this group can be low shrubs or trees up to 20 m tall, showing a remarkable variability in vegetative characters: leaf lengths vary from ca. 5 to 20 cm, their shape from almost circular to narrowly lanceolate and their pubescence from moderately dense to completely absent; if hairs are present, they are always short and appressed and, abaxially, the leaves are often ± waxy; the inflorescences can also vary greatly in length, degree of branching and pubescence, but the flower structure is relatively uniform; the diameter of the flowers is between 5 and 9 mm, the anthers are almost rectangular, usually hardly wider at the base than at the apex and generally longer than wide; the length of the filaments varies from about 1/3 the length of the anthers to slightly longer than the anthers; for the relatively large flowers, the receptacle is very small, often barely sunken; the ovary is usually ± hairy; in male flowers, the sterile ovary (pistillode) always bears a well-developed stigma (Rohwer 1986, Quinet 2008). Regarding distinguishing between a sterile pistillode and a fertile pistil, in dioecious *Ocotea* (subg. *Oreodaphne* Nees sensu Mez (1889); Coe-Teixeira (1980)), both sexes are present in the flower; that is, in male plants, the flowers have rudimentary ovaries that, although somewhat obsolete and sterile, may have their shape more or less normal. Additionally, in female plants, there are stamens that do not bear pollen or bear sterile pollen (Bernardi 1962). As pointed out by van der Werff (2017), the development of the pistillode in staminate flowers of *Ocotea* species can display significant morphological differences, varying from almost fully developed, with a clearly swollen base and a well-developed stigma, to absent (Rohwer 1993; van der Werff 2017). In *Ocotea vervloetii*, its dissected pistillodes clearly lack a developed locule and ovule, indicating a functionally male flower that cannot produce fruit. The stipitiform pistillode of *Ocotea vervloetii* resembles those drawn by Rohwer from dissected male flowers of, for instance: the holotype of *Laurus floribunda* Sw., *Swartz s.n.* (https://www.fundus.uni-hamburg.de/en/collection_records/123716#pfad), isotype of *O. arenaensis* Brooks, *Jean Pierre 12154* (https://www.fundus.uni-hamburg.de/en/collection_records/123965#pfad), holotype of *O. bradei* Coe-Teix., *Brade 7250* (https://www.fundus.uni-hamburg.de/en/collection_records/123996#pfad), syntype of *O. gracilipes* Mez, *Hassler 9165a* (https://www.fundus.uni-hamburg.de/en/collection_records/124077#pfad), syntype of *O. martiana* Mez, *Glaziou 6666* (https://www.fundus.uni-hamburg.de/en/collection_records/124125#pfad), paratype of *O. pulchra* Vattimo-Gil, *Reitz & Klein 1815* (https://www.fundus.uni-hamburg.de/en/collection_records/124200#pfad) and holotype of *Persea lancifolia* Schott, *Schott s.n.* (https://www.fundus.uni-hamburg.de/en/collection_records/124416#pfad).

The *Ocotea floribunda* group *sensu* Rohwer (1986) includes the following species: *Ocotea bragae* Coe-Teix., *O. caniflora* Mez (= *O. floribunda*), *O. floribunda* (Sw.) Mez, *O. glaziovii* Mez, *O. hypoglauca* (Nees & Mart.) Mez, *O. lancifolia* (Schott) Mez s.l. (including *O. kostermansiana* Vattimo-Gil, *O. pulchra* Vattimo-Gil and *O. variabilis* (Nees) Mez in the synonymy, which is not accepted here), *O. percoriacea* Kosterm. and *O. silvestris* Vattimo-Gil. According to Rohwer’s key (Rohwer 1986) for the *Ocotea floribunda* group, *O. vervloetii* keys to *O. hypoglauca* via couplets 1b (largest leaves usually less than 5 cm wide), 3b (flowers stalked…) and 4a (leaf margin strongly rolled downwards along its entire length…). However, the morphological similarities shared between *O. vervloetii* and *O. hypoglauca* are mainly due to both having leaves with revolute margins, which differentiates *O. hypoglauca* from the other species in Rohwer’s key. Despite that, the many differences between them clearly indicate that they belong to different taxa: *O. hypoglauca* is a shrub 3–4.5 m tall of Campos Rupestres of Minas Gerais, whereas *O. vervloetii* is a tree 9–13 m tall of the Montane Dense Ombrophilous Forest of Espírito Santo. The former species has terminal buds glabrous (vs. densely strigulose to sericeous), petiole very short, 0.1 cm long, glabrescent (vs. 0.3–0.7 cm long, glabrescent to sparsely strigulose), leaves lanceolate to elliptic-lanceolate, 5–14 cm × 1.5–4 cm (vs. oblong-obovate to oblanceolate, 2.3–7.1 cm × 0.8–2.4 cm), adaxial surface minutely pitted (vs. strongly bullate), abaxial surface glabrous, glaucous (vs. sparsely to densely strigulose, papillose), 8–12 secondary veins on each side, diverging at an angle of 50º–70º from the mid-rib (vs. 6–8 secondary veins, diverging at 41°–80°), inflorescences 7–17 cm long, glabrescent (vs. 0.6–8.5 cm long, densely strigulose to tomentose), male flowers sparsely golden-tomentose to glabrescent (vs. densely strigulose to tomentose) (Mez 1889, Quinet 2008). Further characters are presented in Table 1.

Regarding the other species in the *Ocotea floribunda* group, the vegetative differences between them and *O. vervloetii* are much more pronounced and will not be discussed here. Further information on descriptions and illustrations can be found elsewhere: for *Ocotea bragae* in Coe-Teixeira (1980), Baitello and Marcovino (2003), Quinet (2008) and Brotto (2018); for *O. floribunda* in Mez (1889), Burger and van der Werff (1990), Rohwer and Schmidt (2014), Moraes (2018) and van der Werff (2025); for *O. glaziovii* in Mez (1889), Vattimo-Gil (1966), Coe-Teixeira (1980), Baitello and Marcovino (2003), Moraes and Oliveira (2007), Quinet (2008), Barbosa et al. (2012), Brotto (2018), Ribeiro (2019) and Moraes and Vergne (2019); for *O. kostermansiana* in Vattimo-Gil (1966), Quinet (2008), Ribeiro (2019) and Moraes and Vergne (2019); for *O. lancifolia* in Coe-Teixeira (1980), Baitello and Marcovino (2003), Moraes and Oliveira (2007), Quinet (2008), Barbosa et al. (2012), Ribeiro (2019) and Moraes and Vergne (2019); for *O. percoriacea* in Mez (1889), as *O. rigida* (Meisn.) Mez, nom. illegit., Quinet (2008) and Ribeiro (2019); for *O. pulchra* in Vattimo-Gil (1956), Baitello and Marcovino (2003), Quinet (2008) and Brotto (2018); for *O. silvestris* in Vattimo-Gil (1958), Baitello and Marcovino (2003), Quinet (2008), Barbosa et al. (2012) and Brotto (2018); and for *O. variabilis* in Mez (1889), Quinet (2008) and Ribeiro (2019).

## Supplementary Material

XML Treatment for Ocotea vervloetii

## Figures and Tables

**Table 1.** Comparative morphology of *Ocotea vervloetii* P.L.R.Moraes & L.S.Santos and *O. hypoglauca* (Nees & Mart.) Mez.

**Characters**	***Ocotea vervloetii***	***Ocotea hypoglauca***
Habit	tree 9–13 m	shrub 3–4.5 m
Branchlet indument	glabrous to sparsely strigulose	glabrous
Terminal bud indument	densely strigulose to sericeous	glabrous
Petiole length (cm)	0.3–0.7	0.1
Petiole indument (adult leaf)	glabrescent to sparsely strigulose	glabrescent
Leaf shape	oblong-obovate to oblanceolate	lanceolate to elliptic-lanceolate
Adaxial leaf surface	strongly bullate	minutely pitted (scrobiculate)
Indument on abaxial leaf surface	sparsely to densely strigulose, papillose	glabrous, glaucous
Leaf length (cm)	2.3–7.1 × 0.8–2.4	5–14 × 1.5–4
Leaf consistency	coriaceous	rigid-coriaceous
Leaf margin	strongly revolute	strongly revolute
Reticulation on adaxial surface	dense	dense
Venation	pinnate	pinnate
Number of secondary veins	6 to 8 on each side	8 to 12 on each side
Angle secondary vein makes with the mid-rib	41°–80°	50º– 70º
Venation pattern	eucamptodromous-brochidodromous	camptodromous-brochidodromous
Inflorescence length (cm)	0.6–8.5	7–17
Flowers per inflorescence	up to 35	up to 50
Male flower diameter (mm)	7–8	2.5–2.8
Male flower indument	densely strigulose to tomentose	sparsely golden-tomentose to glabrescent
Male flower pedicel length (mm)	0.8–1.8	1–4
Male flower receptacle shape	flat, small, shallow, bowl-shaped	small, shallow, bowl-shaped
Male flower receptacle indument inside	densely pubescent	glabrous
Length of filaments of stamens of whorls I & II (mm)	(0.7)1–1.6	0.6–0.68
Length of anthers of whorls I & II (mm)	1.2–1.3(1.5)	0.1–0.12
Length of filaments of stamens of whorl III (mm)	0.9–1.4	0.6–0.68
Length of anthers of whorl III (mm)	1.2–1.5	0.1–0.12
Staminodes of whorl IV	3 or absent	absent
Pistillode	slender, subelliptic	filiform
Distribution	Montane Dense Ombrophilous Forest of Espírito Santo State	Campos Rupestres of Minas Gerais State
